# Polymorphisms in gene encoding TRPV1-receptor involved in pain perception are unrelated to chronic pancreatitis

**DOI:** 10.1186/1471-230X-9-97

**Published:** 2009-12-24

**Authors:** Aura AJ van Esch, Mark P Lamberts, René HM te Morsche, Martijn GH van Oijen, Jan BMJ Jansen, Joost PH Drenth

**Affiliations:** 1 Department of Gastroenterology & Hepatology, Radboud University Nijmegen Medical Centre, Geert Grooteplein 8, P.O. Box 9101, 6500 HB Nijmegen, The Netherlands

## Abstract

**Background:**

The major clinical feature in chronic pancreatitis is pain, but the genetic basis of pancreatic pain in chronic pancreatitis is poorly understood. The transient receptor potential vanilloid receptor 1 (*TRPV1*) gene has been associated with pain perception, and genetic variations in *TRPV1 *may modify the presence and phenotype of chronic pancreatitis. The aim of our study was to investigate the genetic variation of *TRPV1 *in Dutch patients with chronic pancreatitis and healthy controls.

**Methods:**

We genotyped 4 SNPs (rs222749, rs222747, rs224534 and rs8065080) in 228 chronic pancreatitis-patients and 207 healthy controls by PCR, followed by restriction-fragment-length-polymorphism analysis and DNA sequencing. We generated 27 diplotypes and compared prevalence between patients and controls.

**Results:**

There was no significant difference in allele frequency of the 4 *TRPV1 *gene SNPs in patients with chronic pancreatitis and healthy controls. Distribution of diplotypes was not statistically significantly different between patients and controls.

**Conclusion:**

*TRPV1 *diplotypes are not associated with chronic pancreatitis.

## Background

Chronic pancreatitis is an inflammatory process that leads to a progressive and irreversible destruction of the pancreatic parenchyma. The major presenting clinical feature is abdominal pain [1]. Some 60-100% of patients report abdominal pain at a given time during the course of their disease, with pain duration varying from intermittent to persistent and pain intensity ranging from mild to disabling [2]. Consequently, patients with chronic pancreatitis experience substantial impairments in health-related quality of life [3]. The mechanism of pancreatitis-induced pain is unknown [1].

The inter-individual differences in the response of pain suggest that genetic factors may be involved [2,4,5]. A few studies suggest the transient receptor potential vanilloid receptor 1 (*TRPV1*) gene might be involved in pancreatitis [6-9]. The TRPV1 receptor is a nonselective calcium permeant cation channel that belongs to the transient receptor potential family (TRP). It is expressed predominantly in nociceptors that participate in the detection of noxious chemical and thermal stimuli in the dorsal root ganglia and peripheral sensory nerve endings [6,7]. Capsaicin, red pepper, is its natural agonist. Activation of TRPV1 on neurons cause the release of pro-inflammatory neuropeptide substance P and calcitonine G related peptide (CGRP) in the dorsal horn of the spinal cord which is critical for transmitting pain signals from the periphery to the central nervous system [7]. Substance P then binds to the neurokinin-1 receptor (NK1-R) on endothelial cells and promotes extravasation of plasma and proteins into the interstitial tissue and neutrophil infiltration, a process called neurogenic inflammation [10]. There is evidence that activation of TRPV1 is implicated in chronic pancreatitis. In a cerulein-induced pancreatitis model, activation of TRPV1 on sensory neurons promoted neurogenic pancreatic inflammation [10]. This effect was blocked by administration of the selective TRPV1 antagonist capsazepine. In another experimental chronic pancreatitis model, pancreatic TRPV1 receptor mediated inward currents have shown to be greatly enhanced. Moreover, systemic administration of the TRPV1 antagonist SB-366791 markedly reduced both visceral pain behavior and referred somatic hyperalgesia in rats with chronic pancreatitis, but not in control animals [6].

**Figure 1:**
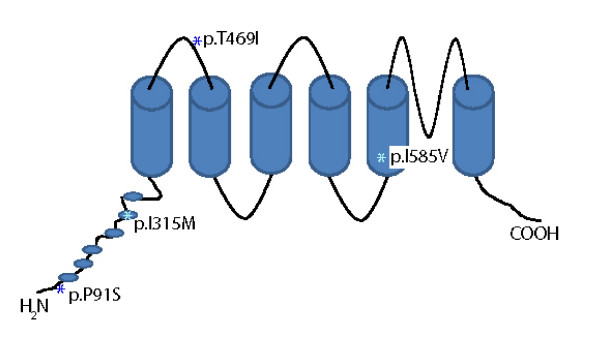
**The TRPV1 gene**. Genomic organization of the TRPV1 gene with genomic structures, positions of splice junction sites. p.P91S (rs222749), p.I315 M (rs222747). p.T469I (rs224534) and p.I585V (rs8065080).

*TRPV1 *is localized on chromosome 17, and consists of 16 exons [11]. HapMap analysis reveals that there are at least 8 non-synonymous single nucleotide polymorphisms (SNPs) that have a heterozygosity rate that exceeds 10%. At least 4 SNPs affect structural domains of *TRPV1*: p.P91S (rs222749) affects the intracellular amino terminus of *TRPV1*; p.I315 M (rs222747) is localized in the ankyrin repeat-containing domain which is predicted to play a role in mediating protein-protein interactions and homotetramerization of the channel; p.T469I (rs224534) is predicted to be located in the extracellular loop between membrane-spanning helices 1 and 2; and p.I585V (rs8065080) is predicted to reside within membrane-spanning helix 5 and affects the transmembrane domain which confers responsiveness to capsaicin (Figure 1). Apart from structural changes, at least 2 SNPs (p.P91S, p.I315 M) modify the functional properties of the channel and induce increased TRPV1 protein expression due to an increased copy number [6].

Collectively, these data suggest a role for TRPV1 in chronic pancreatitis, and we hypothesized that TRPV1 could act as a modifier for chronic pancreatitis and that therefore genetic variations in *TRPV1 *could modify the presence and/or the phenotypical expression of chronic pancreatitis. The aim of our study was to investigate the genetic variation of *TRPV1 *in Dutch chronic pancreatitis patients and healthy controls.

## Methods

### Subjects

We enrolled patients diagnosed with chronic pancreatitis, who visited the outpatient clinic of the Department of Gastroenterology and Hepatology of the Radboud University Nijmegen Medical Centre in the Netherlands between 1999 and 2008. The clinical diagnosis of chronic pancreatitis was based on one or more of the following criteria: presence of typical complaints (recurrent upper abdominal pain, radiating to the back, relieved by leaning forward or sitting upright and increased after eating), suggestive radiological findings, such as pancreatic calcifications or pseudo cysts, and pathological findings (pancreatic ductal irregularities and dilatations) revealed by endoscopic retrograde pancreaticography or magnetic resonance imaging of the pancreas before and after stimulation with secretin. We collected data with special emphasis on the cause of chronic pancreatitis. We specified for the cause of pancreatitis. Patients who had an estimated intake of alcohol of more than 60 g (females) or 80 g (males) daily for more than two years were classified as chronic pancreatitis of alcoholic origin. Hereditary pancreatitis was defined when chronic pancreatitis was present in two or more family members [12]. Idiopathic pancreatitis was diagnosed when precipitating factors such as alcohol abuse, bile stones, trauma, medication, infection, metabolic disorders, and a positive family history were absent. Patients with other causes of pancreatitis, such as anatomic or tropical, were classified as miscellaneous causes.

### Ethics

The study was conducted in accordance with the principles of the Helsinki Declaration and was approved by the local medical ethics review committee. All subjects gave their informed consent.

### Genotyping

For genotyping, a venous blood sample from each subject was collected. Genomic DNA was extracted from peripheral blood leukocytes by standard techniques. We selected 4 non-synonymous *TRPV1 *gene SNPs with a heterozygosity rate exceeding 10%. Primers flanking the SNPs were designed on the basis of publicly available nucleotide sequence of human *TRPV1 *(Additional file 1). Amplification was performed in a 25 μl mixture containing 200 ng of genomic DNA 10 mM Tris-HCl (pH 9.0), 50 mM KCl, 0.1% Triton X-100, 0.25 mM dNTPs, 0.20 μM each primer, 2.5 U Taq polymerase, and 1.5 mM MgCl_2_, except the mixture of rs224534 which contained a concentration of 2.0 mM MgCl_2_. The protocol consists of an initial denaturation at 95°C for 4 minutes, followed by 40 cycles of denaturation at 95°C for 30 seconds, annealing at a particular temperature for each exon for 30 seconds as shown in Additional file 1, and extension at 72°C for 30 seconds, followed by a final extension at 72°C for 7 minutes.

To detect SNP rs222749 (c.271C>T) PCR products were digested with Sau96I (New England Biolabs, Ipswich, USA) resulting in the following fragments: C/C, 322 + 99 bp; C/T, 421 + 322 + 99 bp; T/T, 421 bp. SNP rs222747 (c.945C>G) was detected using BsaBI (New England Biolabs, Ipswich, USA) digestion of the PCR products resulting in the following fragments: C/C, 269 + 162 bp; C/G, 431 + 269 + 162 bp; G/G, 431 bp. To detect SNP rs224534 (c.1406C>T) PCR products were digested with BsrI (New England Biolabs, Ipswich, USA) resulting in the following fragments: C/C, 191 + 115 + 21 bp; C/T, 191 + 136 + 115 + 21 bp; T/T, 191 + 136 bp.

All digested products were subjected to electrophoresis on a 3.5% pronarose MS-8 gel (Hispanagar, Burgos, Spain) and detected by ethidium bromide.

SNP rs8065080 (c.1753A>G) was detected by direct sequencing and analysed by the sequence facility of the Radboud University Nijmegen Medical Centre in the Netherlands (Additional File 2).

### Statistical analysis

Frequency tables were provided for the distribution of the 4 studied SNPs and compared between cases and controls by Pearson's chi-squared test (two-sided Fisher's exact test was used in case values in any of the cells within the table was below 10). We tested for Hardy-Weinberg equilibrium (HWE) among controls using a calculator available on the internet [13].

Diplotypes were generated based on the 4 studied SNPs, missing genes were imputed. The relative risk associated with rare alleles was estimated as an odds ratio (OR) with a 95% confidence interval (CI) with the most common diplotype as a reference. For diplotypes that were only present in either the patient population or healthy controls, no odds ratios were calculated.

We used Haploview 4.0 software to construct a figure of the linkage disequilibrium (LD) plot [14].

## Results

### Characteristics of patients and controls

Samples from a total of 435 subjects were included in our study cohort. The clinical characteristics of the patients and controls are shown in Table 1. The cohort of 228 patients with chronic pancreatitis included 146 male and 82 female patients and the mean age was 47 years (range 17-78 years). 42% of the patients had alcohol related chronic pancreatitis. There were 207 healthy, unrelated controls (79 male and 128 female) with a mean age of 39 (range 18-86 years). In the control group, participants were significantly more often of female gender than in the patient group and they were younger of age.

**Table 1:** Demographic and clinical characteristics of chronic pancreatitis patients and healthy controls

	Patients	Controls	*P *value
n	228	207	
Age (mean, range)	47 (17-78)	39 (18-86)	p = 0.002
Sex (male: female)	146:82	79:128	p < 0,001
Cause of chronic pancreatitis			
Alcoholic	96 (42,1%)		
Hereditary	14 (6,1%)		
Idiopathic	100 (43,9%)		
Miscellaneous	18 (7.9%)		

### SNP

The allele frequencies of the 4 *TRPV1 *gene SNPs in chronic pancreatitis patients and healthy controls are shown in Table 2. There was no evidence that the genotype frequencies of the 4 SNPs among the chronic pancreatitis patients and healthy controls deviated from those expected under Hardy-Weinberg equilibrium (P > 0.05), except for one SNP (rs8065080) in chronic pancreatitis patients. There was no significant difference in allele frequency between chronic pancreatitis patients and healthy controls.

**Table 2:** Allele frequencies of the 4 TRPV1 gene SNPs in chronic pancreatitis patients and healthy controls.

	Alleles		Patients	Controls	*P *value
rs222749					0.251
	C/C	wildtype	204 (89.5%)	189 (92.6%)	
	C/T	heterozygote	24 (10.5%)	15 (7.4%)	
	T/T	homozygote	0	0	
rs222747					0.946
	C/C	wildtype	118 (52%)	106 (51.2%)	
	C/G	heterozygote	86 (37.9%)	78 (37.7%)	
	G/G	homozygote	23 (22.0%)	23 (11.1%)	
rs224534					0.135
	C/C	wildtype	83 (39.1%)	65 (39.4%)	
	C/T	heterozygote	91 (42.9%)	82 (49.7%)	
	T/T	homozygote	38 (17.9%)	18 (10.9%)	
rs8065080					0.512
	A/A	wildtype	85 (38.2%)	68 (35.0%)	
	A/G	heterozygote	86 (38.7%)	86 (44.3%)	
	G/G	homozygote	51 (23.0%)	40 (20.6%)	

### Diplotype analysis

**Figure 2:**
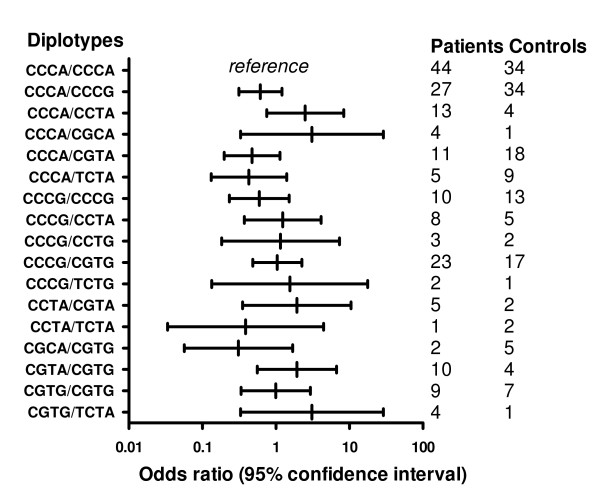
**Diplotype distribution of chronic pancreatitis patients and healthy controls**. Distribution of diplotypes in patients with chronic pancreatitis and healthy controls. The diplotypes are compared to the most prevalent diplotype CCCA/CCCA (reference).

**Figure 3:**
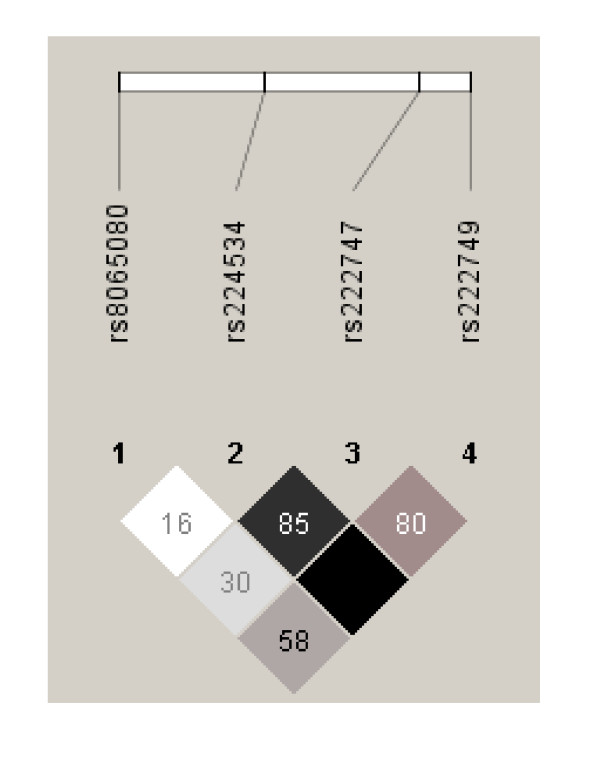
**LD plot D'/LOD**. Linkage disequilibrium (LD) plot across the TRPV1. The box at the top indicates the TRPV1 gene with the 4 investigated SNP's. The LD plot is based on the measure of D'. Each diamond indicates the pair wise magnitude of LD, with dark grey diamonds indicating strong LD (D' > 0.8) and light grey: uninformative. LD: linkage disequilibrium is the non-random association of alleles at two or more loci, not necessarily on the same chromosome. Linkage disequilibrium describes a situation in which some combinations of alleles or genetic markers occur more or less frequently in a population than would be expected from a random formation of haplotypes from alleles based on their frequencies.

Based on the SNP distribution, 27 diplotypes (Additional file 2 and Additional file 3) were generated which were present in chronic pancreatitis patients and in controls, of which 17 diplotypes were present in both groups. Diplotype CCCA/CCCA is most prevalent in both groups. This was therefore considered as the reference in calculating the odds ratios for the remaining diplotypes. Distribution of diplotypes was not statistically significantly different between patients and controls (Figure 2 and 3).

## Discussion and conclusion

We considered genetic variants of *TRPV1 *as a candidate gene for chronic pancreatitis, as we hypothesized that *TRPV1 *could act as a modifier for chronic pancreatitis and that therefore genetic variations in *TRPV1 *could modify the presence and the phenotypical expression of chronic pancreatitis. In this study, we investigated the association between 4 SNPs in the *TRPV1 *gene and chronic pancreatitis in a large cohort of adult chronic pancreatitis patients. We found that the genotypic distribution of none of these 4 SNPs was significantly different between the chronic pancreatitis and control group. Furthermore, based on the SNP distribution 17 diplotypes were generated. There was no significant difference in distribution of diplotypes between patients and controls.

Our study seems to accord with data from TRPV1 knockout animals. There, mice lacking TRPV1 were not protected against pancreatic inflammation induced by cerulein. Because the knockout mice lack functional TRPV1, it seems likely that they developed an alternate inflammatory response pathway that compensates for the loss of TRPV1 signaling. However, compensation by other receptors in this model could not be excluded. The investigators addressed TNFα as a potential candidate for the compensatory response. This suggests that the role of TRPV1 in the generation of pancreatitis is smaller than anticipated on experimental data from other studies [15]. We should remember that this is a study on an acute pancreatitis model that does not reflect chronic pancreatitis. The pathogenesis in acute pancreatitis is probably to some extent dissimilar from chronic pancreatitis. Therefore, one cannot extrapolate results from a secretagogue-induced acute pancreatitis to chronic pancreatitis.

So far, the majority of case-control studies has focused on the association between various *TRPV1 *gene SNPs, most often rs222747 and rs8065080, and pain. One study failed to identify a significant association between these SNPs and cold/heat pain sensitivity in European Americans [16]. Few studies have investigated the role of TRPV1 gene and human chronic pancreatitis. Further credibility to the role of TRPV1 is provided by histochemistry studies showing that TRPV1 is significant upregulated in human chronic pancreatitis and in pancreatic cancer in comparison with patients with a normal pancreas. However, TRPV1 expression was related to the intensity of pain reported by cancer patients, but not to the intensity of pain reported by chronic pancreatitis patients [17].

In our study, we investigated if TRPV1 polymorphisms are associated with chronic pancreatitis, but we were actually interested in the question whether "TRPV1 polymorphisms are associated with pain in patients with chronic pancreatitis?". Our chronic pancreatitis group consisted of patients experiencing pain varying from intermittent to persistent and pain intensity ranging from disabling to no pain or mild pain. We did not directly quantify pain, which makes it difficult to study the exact correlation between TRPV1 and pain due to chronic pancreatitis in this population. It is very complex to investigate pain, due to different levels of pain that patients experience, the use of analgetic drugs and different pain scales. Moreover, there are several confounding variables, such as dependence of analgetic drugs and the use of alcohol or other narcotic agents. However, since almost every patient with chronic pancreatitis will experience pain during the course of their disease, we lumped patients and investigated TRPV1 in chronic pancreatitis patients from our cohort.

Xu and colleagues investigated functional effects of nonsynonymous SNPs in the human *TRPV1 *gene [18]. They found that polymorphisms rs222747 and perhaps rs222749 resulted in markedly increased abundance of the variant TRPV1 protein at the level of whole-cell expression, and at the level of expression at the cell surface. The increment in rs222747 mRNA level was probably not sufficient to account for the marked change in protein expression.

Our study does have certain limitations. First, with our current sample size, we were able to detect a 10 percent difference with 80% power and a 0.05 two-sided significance level. As a consequence, we cannot rule out the existence of a smaller than 10% difference between patients with pancreatitis and controls. The clinical relevance of a difference below 10% is rather low. Moreover, we combined 4 SNPs into diplotypes, making the study even more robust excluding chance results.

Second, we have no insights in alcohol use in our healthy controls. Recently other investigators found that the TRPV1 receptor is activated by ethanol and has a role in specific behavioral effects of ethanol [19]. Since a large proportion of chronic pancreatitis patients developed chronic pancreatitis as a result of liberal alcohol use, these variables may likewise be important.

In conclusion, our results suggest that these four SNPs in do not seem to modify the presence and phenotypical expression of chronic pancreatitis.

## Competing interests

The authors declare that they have no competing interests.

## Authors' contributions

AAJvE, MPL, JBMJJ, MGHvO and JPHD participated in the design of the study and drafted the manuscript. MPL and RHMtM carried out the collection of the data and the genotyping. AAJE and MGHvO carried out the statistical analysis. RHMtM designed the PCR procedure and gave technical advice on the genotyping. All authors read and approved the final manuscript.

## Pre-publication history

The pre-publication history for this paper can be accessed here:

http://www.biomedcentral.com/1471-230X/9/97/prepub

## Supplementary Material

Additional file 1 PCR oligonucleotide primers used in this study. Click here for file

Additional file 2 **TRPV1 polymorphisms**. Identification of *TRPV1 *polymorphisms using RFLP or sequencing. Electrophoresis pattern of digested PCR products for: SNP rs222749 (A) lane 1: C/T, 2:C/C, 3:C/C, 4:C/C, 5: C/T and lane 6:C/T; SNP rs222747 (B) lane 1: C/C, 2:C/G, 3:C/G, 4:G/G, 5: C/C and lane 6:G/G; SNP rs224534 (C) lane 1: C/C, 2:T/T, 3:T/T, 4:C/C, 5: C/T and lane 6:C/C. D) Electrospherogram of a heterozygous sample for SNP rs8065080. Click here for file

Additional file 3 Diplotype distribution of chronic pancreatitis patients and healthy controls. Click here for file
