# Supplementary material for: Polymorphisms in gene encoding TRPV1-receptor involved in pain perception are unrelated to chronic pancreatitis
Source: BMC Gastroenterol. 2009 Dec 24;9:97. doi: 10.1186/1471-230X-9-97 (PMC2813232; doi:10.1186/1471-230X-9-97)
Supplement: Additional file 1 — PCR oligonucleotide primers used in this study. [file 1471-230X-9-97-S1.DOC]

| SNP number | Allele | Annealing temperature | Primer | Sequence |
| --- | --- | --- | --- | --- |
| rs222749 | T:C | 57 ºC | TRPV1Ex2F | 5’-CGGCGTGGTGGCTGCTGCA-3’ |
|  |  |  | TRPV1Ex2R | 5’-TAGCCCAGAAGCCAGACCAC-3’ |
| rs222747 | C:G | 60 ºC | TRPV1Ex6F | 5’-AGTTTGGAGGCCGGTGGTTC-3’ |
|  |  |  | TRPV1Ex6R | 5’-TCCTCTCCCATGCCATCAGC-3’ |
| rs224534 | A:G | 55 ºC | TRPV1Ex9F | 5’-GGCAGGGACTATGGCTTCA-3’ |
|  |  |  | TRPV1Ex9R | 5’-CTCATCTTCACCTCTGCGTC-3’ |
| rs8065080 | C:T | 57 ºC | TRPV1Ex12F | 5’-GCCCTTCCCTCAGCTCCTCC-3’ |
|  |  |  | TRPV1Ex12R | 5’-CAGCTCCTGGCAGAGTCTTCA-3’ |
