# Supplementary figures and images for: Polymorphisms in gene encoding TRPV1-receptor involved in pain perception are unrelated to chronic pancreatitis
Source: BMC Gastroenterol. 2009 Dec 24;9:97. doi: 10.1186/1471-230X-9-97 (PMC2813232; doi:10.1186/1471-230X-9-97)

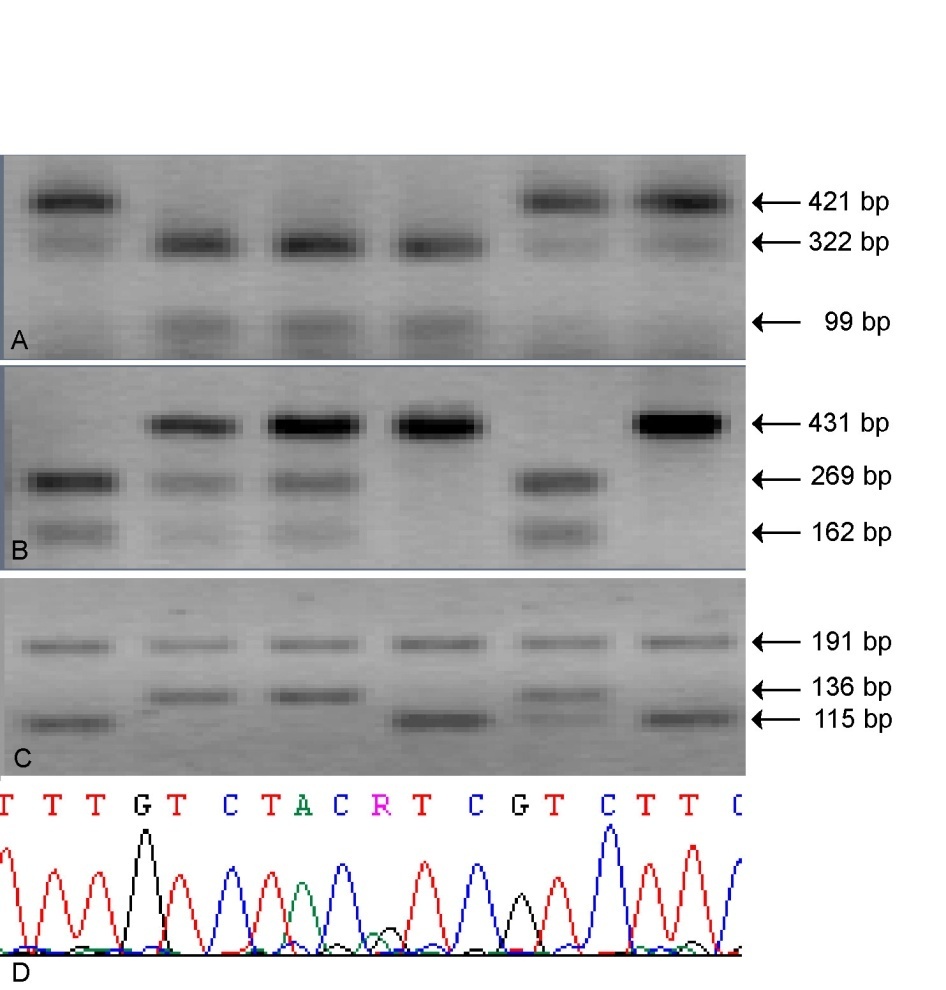

Supplement: Additional file 2 — TRPV1 polymorphisms. Identification of TRPV1 polymorphisms using RFLP or sequencing. Electrophoresis pattern of digested PCR products for: SNP rs222749 (A) lane 1: C/T, 2:C/C, 3:C/C, 4:C/C, 5: C/T and lane 6:C/T; SNP rs222747 (B) lane 1: C/C, 2:C/G, 3:C/G, 4:G/G, 5: C/C and lane 6:G/G; SNP rs224534 (C) lane 1: C/C, 2:T/T, 3:T/T, 4:C/C, 5: C/T and lane 6:C/C. D) Electrospherogram of a heterozygous sample for SNP rs8065080. [file 1471-230X-9-97-S2.DOC]
