# Supplementary material for: Polymorphisms in gene encoding TRPV1-receptor involved in pain perception are unrelated to chronic pancreatitis
Source: BMC Gastroenterol. 2009 Dec 24;9:97. doi: 10.1186/1471-230X-9-97 (PMC2813232; doi:10.1186/1471-230X-9-97)
Supplement: Additional file 3 — Diplotype distribution of chronic pancreatitis patients and healthy controls. [file 1471-230X-9-97-S3.DOC]

|  | Patients | Controls |
| --- | --- | --- |
| CCCA/CCCA (reference) | 44 | 34 |
| CCCA/CCCG | 27 | 34 |
| CCCA/CCTA | 13 | 4 |
| CCCA/CGCA | 4 | 1 |
| CCCA/CGCG | 2 | 0 |
| CCCA/CGTA | 11 | 18 |
| CCCA/TCTA | 5 | 9 |
| CCCG/CCCG | 10 | 13 |
| CCCG/CCTA | 8 | 5 |
| CCCG/CCTG | 3 | 2 |
| CCCG/CGCA | 0 | 2 |
| CCCG/CGCG | 2 | 0 |
| CCCG/CGTG | 23 | 17 |
| CCCG/TCTG | 2 | 1 |
| CCCG/TGTA | 1 | 0 |
| CCTA/CCTG | 2 | 0 |
| CCTA/CGTA | 5 | 2 |
| CCTA/TCTA | 1 | 2 |
| CGCA/CGTG | 2 | 5 |
| CGCG/CGTG | 1 | 0 |
| CGTA/CGTA | 0 | 7 |
| CGTA/CGTG | 10 | 4 |
| CGTA/TCTA | 5 | 0 |
| CGTA/TGTA | 1 | 0 |
| CGTG/CGTG | 9 | 7 |
| CGTG/TCTA | 4 | 1 |
| CGTG/TCTG | 1 | 0 |
